# Cochrane: the unfinished symphony of research synthesis

**DOI:** 10.1186/s13643-016-0290-9

**Published:** 2016-07-14

**Authors:** Ian Roberts, Katharine Ker

**Affiliations:** Clinical Trials Unit, London School of Hygiene & Tropical Medicine, Keppel Street, London, WC1E 7HT UK

## Abstract

The NHS needs valid information on the safety and effectiveness of healthcare interventions. Cochrane systematic reviews are an important source of this information. Traditionally, Cochrane has attempted to identify and include all relevant trials in systematic reviews on the basis that if all trials are identified and included, there should be no selection bias. However, a predictable consequence of the drive to include all trials is that some studies are included that are not trials (false positives). Including such studies in reviews might increase bias. More effort is needed to authenticate trials to be included in reviews, but this task is bedevilled by the enormous increase in the number of ‘trials’ conducted each year. We argue that excluding small trials from reviews would release resources for more detailed appraisal of larger trials. Conducting fewer but broader reviews that contain fewer but properly validated trials might better serve patients’ interests.

## Background

The UK National Institute for Health Research (NIHR) provides public funding for systematic reviews because the information they provide informs healthcare decision making in the NHS and the commissioning of new research. For both purposes, it is essential that the information reviews provide is valid and up-to-date. In October 2015, NIHR announced an evaluation of its investment in Cochrane infrastructure for the production and dissemination of reviews. It will consider the health and economic impact of Cochrane reviews by ‘assessing the quantity, quality and impact of reviews on policy, practice and research’. Of the parameters assessed (quantity, quality, impact), quality is the most important. Systematic reviews will not bring health and economic benefits if their conclusions are misleading. We consider the main threats to validity in systematic reviews and how they can be minimised cost-effectively.

The conclusions of a systematic review can be misleading if it includes a biased sample of trials (selection bias in study identification and inclusion) and/or the effect estimates in the included trials are biased. Validity requires an unbiased sample of unbiased effect estimates. Neither criterion is easy to satisfy. About half of all trials are not published, and the results of published and unpublished trials differ systematically [1, 2]. Even if all trials could be identified, selective reporting of outcomes remains an important source of bias [3]. Because of poor methodology, the results of many trials are biased, and incomplete or inaccurate reporting of trial methods frustrates quality assessment.

## Find all the trials

Since its inception, Cochrane has attempted to avoid selection bias in study identification and inclusion by conducting exhaustive searches for all trials, published or unpublished, irrespective of language of publication. If all trials are included, there should be no such selection bias. A ‘highly sensitive search strategy’ was developed to identify all trials in the main electronic bibliographic databases [4]. Because many trials in indexed journals were not coded as such by the National Library of Medicine, trial identification was supplemented by hand-searching. This resulted in a major initiative to re-tag records that were not properly coded. The Cochrane Central Register of Controlled Trials (CENTRAL) launched in 1996 includes trials identified in electronic databases and by hand-searching [5, 6]. Trial identification has become a highly specialised activity, and almost all Cochrane Groups have a dedicated trial search co-ordinator to undertake this role. However, despite extensive efforts to identify and include all relevant trials, selection bias (in study identification and inclusion) still casts doubt on the conclusions of many reviews.

## The problem of false positives

A predictable consequence of the drive to find and include all trials is that some studies are included that are not trials (false positives). Maximising sensitivity often decreases specificity. Many reports claiming to be ‘randomised trials’ are not in fact randomised, often owing to a lack of an understanding of trial design among the authors and sometimes due to downright deception. An interview study of Chinese authors of so-called randomised controlled trials found that only 7 % were authentic [7]. Even for trials conducted at university-affiliated hospitals, only 56 % were authentic [7]. Although it would be inappropriate to assume that the prevalence of ‘false positives’ is as high in other settings, it would also be inappropriate to ignore these findings and take trials at face value without conducting further checking.

Meta-analyses of baseline variables in systematic reviews of randomised trials often reveal surprising imbalances suggesting that randomisation was either subverted or absent [8, 9]. And some reviews contain fraudulent data. A Cochrane review showing that high-dose mannitol reduced the risk of death after head injury was rewritten after an investigation was unable to confirm that three of the included trials took place [10]. The conclusions of a review of starch solution in critically ill patients changed importantly after excluding seven ‘trials’ from an investigator whose research was retracted due to misconduct [11, 12]. Many journal editors and systematic reviewers take trial reports at face value with little or no effort to confirm whether a trial actually took place or how reliably it was conducted. A recent survey of authors of systematic reviews found that 38 % had no contact with the authors of the original studies [13]. Indeed, the amount of contact with authors is rarely reported in reviews. Checking with ethics committees that ethical approval was obtained for a trial is one of the few ways to obtain independent confirmation that a trial took place and even then is not completely reliable. However, very few (3 %) reviewers check whether the included studies had such approval [13]. Unless sensitive searching is accompanied by similarly rigorous efforts to ensure the integrity of the included trials, the precision gained by including a larger number of ‘trials’ could be outweighed by an increase in bias.

NIHR funding for Cochrane Groups is proportional to the number of reviews published and the number of trials included in reviews. Both criteria have seriously detrimental unintended consequences. First, it incentivises the fragmentation of evidence. For example, there are over 100 Cochrane reviews on the treatment of hypertension, including separate reviews of treatment trials in people with and without diabetes, rather than a single review assessing whether diabetes is an effect modifier. Second, there is a financial incentive to include every study purporting to be a ‘trial’ with no incentive to root out false positives.

## The challenge of validating trials

Unless rigorous efforts to identify trials are accompanied by at least as much effort to assess their integrity and completeness, extensive searching may have the unintended consequence of *reducing* the reliability of reviews. Results from individual patient data meta-analyses are likely to be more reliable in this respect since they involve checking the integrity and completeness of all included data. However, they can be challenging and costly and are not immune from selection bias [14]. Nevertheless, simpler checks are possible. We believe that authors should be required to provide evidence that the trial actually took place and that the participants were properly randomised, that there were no post-randomisation exclusions and no selective reporting. This could be combined with the use of software to detect plagiarism and statistical data checking (e.g. detection of extreme between study homogeneity). Reviews should routinely report their validation strategies, including which trials were confirmed and which authors were unresponsive.

**Fig. 1:**
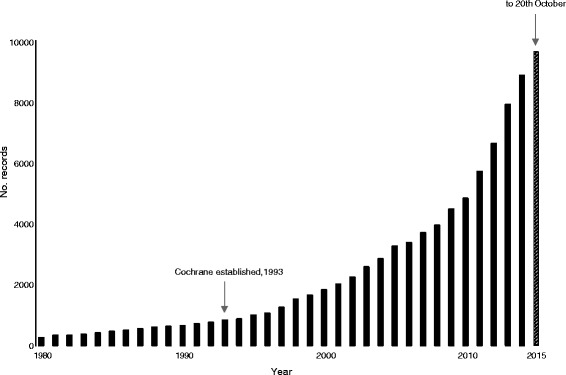
Number of trial records on PubMed by year, 1980 to October 2015

However, the task of identifying and authenticating all potentially relevant trials is bedevilled by the enormous increase in the number of ‘trials’ conducted each year (Fig. 1). In 1993, the year the Cochrane Collaboration was founded, 823 trials were indexed in PubMed. Twenty-two years later, the corresponding figure is 9673 trials. If efforts to prevent bias through the complete ascertainment of trials have failed to date, it is unlikely they will succeed in future without a considerable increase in resources. Many Cochrane reviews are already out-of-date and the situation can only be expected to get worse.

## Small trials—are they worth the effort?

**Fig. 2:**
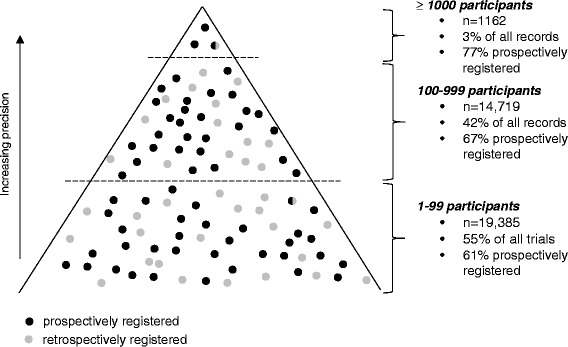
Number of prospectively and retrospectively registered randomised trials of drug interventions started since the introduction of ICMJE’s registration policy in July 2005, stratified by sample size

Most trials are small. Over half of registered trials include fewer than 100 participants (Fig. 2). Many important health outcomes (death, myocardial infarction, stroke, sepsis, pneumonia) are dichotomous and occur in only a small proportion of trial participants. Most small trials register only a few such outcome events in each arm. With such sparse outcome data, there is a substantial risk of selective under-reporting of trials and patient outcomes. This is a major threat to validity. One fifth of Cochrane meta-analyses with a statistically significant result became non-significant after adjusting for outcome reporting bias [3].

Meta-epidemiological studies show that small, single-centre trials generally provide larger estimates of treatment effects than large, multi-centre trials [15–18]. This could be due to strict patient selection and better intervention compliance in small trials. However, it is often due to the poor quality of small trials and their greater risk of selection bias. Furthermore, small trials that are stopped early for apparent benefit often provide implausibly large effects (the overestimates are smaller in large trials) [19, 20]. Random effects models exacerbate small study bias. In the presence of heterogeneity, random effects models give greater weight to small studies, which are more susceptible to bias. Small studies with large effects appear to ‘anchor’ the meta-analysis such that the result is largely unchanged regardless how large the subsequent trials. When meta-analyses are restricted to larger studies, treatment effects that appear large and statistically significant when all trials are combined usually become smaller [16]. This suggests that including all apparent trials in systematic reviews can increase rather than decrease bias.

The well-documented unreliability of small trials offers an opportunity to increase the validity of reviews whilst reducing the burden and cost of conducting and maintaining them. Although large trials provide more information about the treatment effect than small trials, the time and effort required to evaluate a trial is often inversely related to sample size. Limiting inclusion to larger trials would release resources that could be redirected to a more thorough critical appraisal of the trials that provide the most information. The sample size cut-off would clearly depend on the objectives of the review and the outcomes of interest. The information content of a trial depends on the number of events rather than the number of participants. Quantitative research is needed on the sample size cut-off that would provide an optimal balance of sensitivity and specificity, and on other markers of unreliable data.

The exclusion of grossly underpowered trials from systematic reviews and meta-analyses should reduce bias, but it is not a guarantee. A more reliable approach would be to identify trials for inclusion in systematic reviews from trial registers [21]. Systematic reviews that entail complete ascertainment of results from all (or an unbiased sample of) prospectively registered trials (i.e. trials registered before the first patient is enrolled) should not be affected by selection bias in trial identification and inclusion. Indeed, preventing such selection bias is one of the main purposes of registers, and trials with large sample sizes are more likely to be prospectively registered (Fig. 2). However, including only prospectively registered trials is not sufficient. Trials that were prospectively registered but not reported must be sought out and included to avoid bias. Similarly, data on health outcomes that were collected but not fully reported must be pursued and included. This will take time and effort and will be greatly facilitated by excluding the myriad small trials that provide negligible amounts of information.

## Counter arguments

Some argue that including all trials, regardless of size or quality, allows reviewers to draw attention to the scandal of low-quality, underpowered trials [22]. We agree that the main contribution of systematic reviews has been to highlight the miserable unreliability of most biomedical research. However, in the light of the methodological advances from meta-epidemiological studies, it is questionable whether highlighting poor quality remains a legitimate use of public funds. Indeed, including such trials in reviews gives them unwarranted endorsement.

Another argument for including small trials in reviews is that the combined results from small trials often motivate larger high-quality studies. For example, Chalmers and Glasziou argue that ‘funders and regulators cannot be expected to support and endorse large studies without some reassurance from the results of smaller existing studies that the substantial investment needed is justified’ [23, 24]. Whilst we agree that investment in new research should be preceded by systematic assessment of existing evidence, it is essential to avoid making funding decisions that are heavily influenced by biased research. Because effect estimates from systematic reviews often inform sample size calculations, there is a danger that inflated effect estimates from reviews of small trials motivate new trials that are underpowered to detect realistic treatment effects. For example, a Cochrane review of randomised trials of the effect of preoperative statins on the risk of post-operative atrial fibrillation included 17 small trials with a total of 2138 participants and reported a halving of the odds of post-operative atrial fibrillation with statin treatment (OR = 0.54; 95 % CI 0.43 to 0.67; *p* < 0.01) [25]. This claim was later refuted by a randomised trial (the Statin Therapy in Cardiac Surgery (STICS) trial) that included more outcome events (cases of atrial fibrillation) than all the previous trials combined that found no reduction in atrial fibrillation (OR = 1.04; 95 % CI 0.84 to 1.30; *p* = 0.72) [26]. Fortunately, the STICS trial investigators were appropriately sceptical of the large treatment benefits suggested by the Cochrane review of small trials and conducted a trial with sufficient power to exclude a more plausible effect. However, although the new trial appears to refute the conclusion of earlier smaller trials because of the anchoring effect of small studies with large treatment effects, the updated meta-analysis will still suggest a significant reduction in atrial fibrillation with statin treatment, a conclusion that seems unlikely.

## Conclusion

The NHS needs valid information on the safety and effectiveness of healthcare interventions. This information must be provided cost-effectively. Although investment in systematic reviews can be more cost-effective than conducting new trials, this should not rule out consideration of how to improve the cost-effectiveness of conducting reviews. We argue that attempting to identify and include all apparently relevant trials might increase rather than decrease bias and may not be the most cost-effective approach. Many supposed ‘trials’ are not in fact randomised trials. The gulf between individual participant data meta-analyses, where all data is thoroughly checked for accuracy and completeness, and systematic reviews based on published data from apparent trials is too wide. More effort is needed to validate trials and obtain data on unreported outcomes. Excluding grossly underpowered small trials from reviews might increase validity and release resources for more detailed appraisal of included trials. NIHR incentives to conduct narrowly focused reviews including everything purporting to be a trial should be re-considered. Incentives to conduct fewer but broader reviews that contain fewer but properly validated trials might better serve patients’ interests.

## Abbreviations

NHS, National Health Service; NIHR, National Institute for Health Research
